# Colorectal Cancer: From Genetic Landscape to Targeted Therapy

**DOI:** 10.1155/2021/9918116

**Published:** 2021-07-06

**Authors:** Mouade El Bali, Joaira Bakkach, Mohcine Bennani Mechita

**Affiliations:** Biomedical Genomics and Oncogenetics Research Laboratory, Faculty of Sciences and Techniques of Tangier, Abdelmalek Essaâdi University, Tetouan, Morocco

## Abstract

Colorectal cancer (CRC) is the third most common cancer type and the second cause of death worldwide. The advancement in understanding molecular pathways involved in CRC has led to new classifications based on the molecular characteristics of each tumor and also improved CRC management through the integration of targeted therapy into clinical practice. In this review, we will present the main molecular pathways involved in CRC carcinogenesis, the molecular classifications. The anti-VEGF and anti-EGFR therapies currently used in CRC treatment and those under clinical investigation will also be outlined, as well as the mechanisms of primary and acquired resistance to anti-EGFR monoclonal antibodies (cetuximab and panitumumab). Targeted therapy has led to great improvement in the treatment of metastatic CRC. However, there has been variability in CRC treatment outcomes due to molecular heterogeneity in colorectal tumors, which underscores the need for identifying prognostic and predictive biomarkers for CRC-targeted drugs.

## 1. Introduction

Colorectal cancer (CRC) is considered the third most prevalent cancer and the second cause of death by cancer worldwide [1]. In 2018, 1.8 million new CRC cases were reported and 881,000 persons died of the disease, which accounted for 6.1% and 9.2% of new cases and deaths, respectively [2]. An increasing incidence trend of 2.5 million cases has been predicted in 2035 [3].

Currently, the 5-year overall survival (OS) rate of CRC is estimated at 64% for all stages in the United States, and this seems to decrease to nearly 12% for metastatic CRC (mCRC) [4, 5].

Surgery alone or in combination with chemotherapy and radiotherapy in the adjuvant setting remains the main treatment option in cases of early diagnosis, while surgery is no longer effective for advanced stages that represent 25% of CRCs cases [6]. Unfortunately, the efficacy of cytotoxic therapies may be altered by the rapid evolution of drug resistance and the occurrence of cancer recurrence [7]. Hence, developing other treatment options for CRC, especially for mCRC to increase its overall survival and reduce its severity, is highly needed.

With the advancement in our understanding of carcinogenesis mechanisms and the underlying molecular pathways, treatment of CRC, especially mCRC, has evolved considerably over the past years, which was reflected by using many chemotherapy combinations and integrating novel targeted drugs into clinical practice. This advancement in chemotherapy and targeted drugs has led to significantly improve OS to over 40 months for mCRC patients [8].

**Figure 1:**
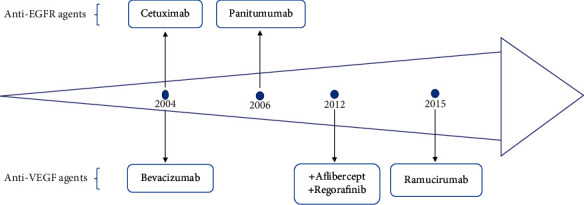
Targeted agent approved for colorectal cancer. EGFR: epidermal growth factor receptor; VEGF: vascular endothelial growth factor.

Cetuximab was the first targeted agent for CRC that has been approved by the Food and Drug Administration (FDA) in 2004, followed by bevacizumab in the same year. Since then, many other targeted drugs for CRC have been brought to market successively (Figure 1). Targeted agents currently used for the treatment of CRC may be divided into three categories: anti-Vascular Endothelial Growth Factor (VEGF) such as bevacizumab, aflibercept, and ramucirumab; anti-Epidermal Growth Factor Receptor (EGFR) antibodies such as cetuximab and panitumumab; and finally multikinase inhibitors like regorafenib [9].

The advancement in understanding molecular pathways involved in CRC carcinogenesis has also led to many molecular classification systems. The Cancer Genome Atlas (TCGA) and the Consensus Molecular Subtype (CMS) are considered the main classifications. CMS classification has been proposed after analyzing the pathological and molecular profile of CRC patients from many studies. In addition to its potential prognostic and predictive value, CMS can also help to explain the CRC heterogeneity caused by genetic and epigenetic mechanisms [10].

The current review aims to provide an overview of molecular pathways involved in CRC carcinogenesis, as well as molecularly defined subtypes and their clinical implication. We will also summarize available and future CRC-targeted agents and discuss anti-EGFR resistance mechanisms.

## 2. Molecular Pathways in CRC

### 2.1. Inherited CRC

The etiologies of CRC are either genetic or environmental or both [11]. CRC is divided into hereditary and sporadic forms, with approximately 75–80% of sporadic forms [12].

Approximately 5% of all CRC cases are caused by inherited germline mutations in some key genes, leading to colorectal carcinogenesis. Approximately 20% of the remaining 95% of CRC cases present a positive family history, which cannot account as a true hereditary form of CRC. Many syndromes have been identified; the most frequent syndromes are Lynch Syndrome (Hereditary Nonpolyposis Colorectal Cancer (HNPCC)) and Familial Adenomatous Polyposis (FAP) [13, 14].

*Lynch Syndrome* (HNPCC) is the most common hereditary CRC syndrome representing 2-3% of all CRC patients [14]. It is an autosomal dominant syndrome, caused by germline mutations in DNA mismatch repair (MMR) genes, which lead to amplifying the replication errors, increasing rate, and the potential of malignancy. These genes included *MLH1*, *MSH2*, *PMS2*, and *MSH6* resulting in microsatellite instability (MSI) when mutated [15].

*Familial Adenomatous Polyposis* (FAP) is an autosomal dominant disorder, which is characterized by colorectal adenomatous polyps, which ranged from hundreds to thousands of polyps [16]. It is caused by germline mutations in the adenomatous polyposis coli (*APC*) gene with a frequency of 1% of all CRCs [14]. *APC* gene is a tumor suppressor gene, coding for a protein that regulates the cytoplasm degradation of *β*-catenin. These two molecules are essential components of the Wnt signaling pathway [17].

There are other rare forms of familial CRC including the *MYH-Associated Polyposis* (MAP), which is an autosomal recessive disorder caused by biallelic mutations in the *MYH* gene. Tumors in this form are commonly microsatellite stable and exhibit a high frequency of APC somatic mutations and a low rate of loss of heterozygosity (LOH) [14]. Additionally, the *Hamartomatous polyposis syndromes* that englobe the syndrome of *PeutzJeghers* (PJS), the syndrome of Juvenile Polyposis (JPS), and the syndrome of Cowden are autosomal dominant syndromes caused by germline mutations in *STK11/LKB1*, *BMPR1A/SMAD4*, and *PTEN,* respectively [18].

More details about these hereditary CRC syndromes, diagnosis, and management approaches have been well reviewed by Kastrinos and Syngal [19].

### 2.2. Sporadic CRC

With respect to sporadic CRC, Fearon et al. have suggested a colorectal carcinogenesis model that correlated specific genetic landscapes with changing tissue morphology, from adenomas to carcinomas [12, 20]. Genomic instability is considered an essential component of this transformation process [14]. There are three main categories of genomic instability in CRC. Chromosomal instability (CIN), the most frequent representing 70–85% of CRCs, is characterized by the accumulation of numerical or structural chromosomal abnormalities (aneuploidy). FAP is the inherited syndrome associated with these changes [21, 22]. Another type of genomic instability is the MSI, which is caused by MMR alteration [22]. With the increasing knowledge with regard to the involvement of epigenetic factors, particularly the promoter sequence methylation, in the development of certain subsets of cancers and polyps, the third pathway of genomic instability has emerged “CpG Island Methylator Phenotype (CIMP+)” [22]. All these alterations are presented in detail hereinafter.

#### 2.2.1. Chromosome Instability

CIN accounts for 65–70% of sporadic CRCs. It is characterized by the high frequency of LOH, subchromosomal genomic amplifications, and extensive chromosome imbalances (aneuploidy). In addition to these karyotypic abnormalities, the accumulation of particular mutations in some specific tumor suppressor genes and oncogenes activates CRC pathogenesis [23]. The main genes which are mutated during this pathway are as follows:

 
*APC* gene: It is a tumor suppressor gene located on chromosome 5q21. APC functional protein plays an important role in regulating differentiation, adhesion, apoptosis, development, migration, and chromosomal segregation. Mutations or loss of this gene have been found in 40–70% of CRCs and were considered the earliest genetic events in colorectal carcinogenesis [24, 25].
  APC protein is a part of a complex that phosphorylates the *β*-catenin, which causes its ubiquitination and destruction in the proteosome. Truncated APC protein destabilizes the complex and increases the cytoplasmic *β*-catenin level, which translocates to the nucleus and activates the transcription of various genes involved in tumor growth and invasion, by interacting with the T-cell factor/lymphoid [24]. Half of CRC cases with intact *APC* have activating mutations in the *β-catenin* gene [26], which reflects the importance of the Wnt pathway.
 
*KRAS* oncogene: The frequency of *KRAS* protooncogene mutations is estimated to range from 30 to 60% in CRCs and large adenomas. Most activating mutations were found to be located in codons 12 and 13 of exon 1 [27]. Activation of K-Ras is known to affect various cellular pathways that regulate cellular growth, survival, proliferation, apoptosis, cytoskeleton organization, cell motility, differentiation, and inflammation [14, 23].
  Activation of the *KRAS* gene has been suggested to play a significant role in the transition from adenoma to carcinoma [28].
 
*SMAD2, SMAD4*, and *DCC*: These three genes are located at chromosome 18q21. The allelic loss of this site has been found in 60% of CRCs [29]. *DCC* gene encodes for a transmembrane receptor that promotes apoptosis, whereas *SMAD2* and *SMAD4* are part of the transforming growth factor-*β* (TGF-*β*) signaling pathway, which regulates growth as well as apoptosis [22, 30].
 
*TP53*: It is located on 17p13.1 and encodes for a tumor suppressor protein p53 whose inactivation is usually a late event in the CRC carcinogenesis process [31]. It is widely known that p53 dysfunction is a universal biomarker of human tumors and the loss of its function has been reported in 4–26% of adenomas, 50% of adenomas with invasive foci, and 50–75% of CRCs, which define its role in the transition from adenoma to carcinoma [23, 32].

The CIN pathway is related to mutations in the *APC* gene or allelic loss at chromosome 5q (*APC, MCC* genes), followed by Kirsten rat sarcoma viral oncogene (*KRAS*) mutation, loss of 18q (*DCC, SMAD2,* and *SMAD4* genes), and finally, deletion of 17p, containing the famous tumor suppressor gene *TP53* [24].

#### 2.2.2. Microsatellite Instability

Microsatellites are short sequences with repeated nucleotides, which are distributed across the entire human genome, and consist of mononucleotide, dinucleotide, or higher-order nucleotide repeats such as (A)n or (CA)n [33]. These microsatellites are especially motifs of mutation accumulations, due to the decreasing of DNA polymerase efficiency. The most common errors associated with microsatellites are base-base mismatches and insertion-deletion loops (IDLs) [33, 34].

MMR systems are charged to maintain genomic stability by identifying and repairing base-pair mismatches that occur during DNA replication. Mutator phenotype accompanied with MSI is a result of the MMR systems' inability to correct these errors. There are at least 7 proteins in the mismatch repair systems: hMLH1, hMLH3, hMSH2, hMSH3, hMSH6, hPMS1, and hPMS2, forming 5 protein dimers, which are the MutS*α* (MSH2, MSH6), MutS*β* (MSH2, MSH3), MutL*α* (MLH1, PMS2), MutL*β* (MLH1, PMS1), and finally MutL*γ* (MLH1, MLH3) [34, 35].

In order to test the MSI, there are two main methods: immunohistochemistry (IHC) which serves for the detection of the expression level of the four main MMR proteins (MSH2, MSH6, MLH1, and PMS2) directly from the tumor tissue. Loss of expression of at least one of these proteins means that the tumor is deficient MMR (dMMR) and as a consequence MSI [36]. The second method is based on testing a DNA microsatellite panel. In this method, MSI-High (MSI-H) is defined when 40% of the markers are unstable [36]. In 1998, a panel of five microsatellite markers called the Bethesda panel has been proposed for the first time by the “International Workshop on Microsatellite Instability and RER Phenotypes in Cancer Detection and Familial Predisposition,” which includes two mononucleotides (BAT25 and BAT26) and three dinucleotides (D5S346, D2S123, and D17S250) [37]. Another panel called the Pentaplex panel, composed of five mononucleotides markers, has been proposed (BAT25, BAT26, NR21, NR24, and NR27), due to the high sensitivity of mononucleotides markers compared to dinucleotides. Based on the Pentaplex panel, two types of tumors have been established: MSI (MSI-High) with at least three unstable markers, and microsatellite stability (MSS) with no instability, or the instability in one marker [36].

Approximately 15% of CRC patients show an MSI, 3% of which are caused by germline mutations (Lynch Syndrome), and 12% are due generally to sporadic hypermethylation in the promoter of the *MLH1* gene [38]. Most studies suggested that MSS tumors had a worse prognosis than those with MSI. Additionally, a strong correlation has been found between sporadic MSI and the existence of V600E *BRAF* mutation [39, 40].

#### 2.2.3. CpG Island Methylator Phenotype

Epigenetic regulation of gene expressions is defined as heritable changes without any alteration in the DNA sequence. These epigenetic changes are found to play an important role in the carcinogenesis of many carcinomas including CRC and offer an explanation of some phenotypes of this disease. Histone modifications or DNA methylation are thought to be the most common cause of epigenetic alterations [41].

CpG island methylation is a typical epigenetic event in colorectal carcinogenesis, accounting for 20% in CRCs [42]. The concept of CIMP in CRC has been originally reported in 1999 by Toyota et al. [43]. It occurs generally by DNA hypermethylation at the 5′-CG-3′ (CpG) dinucleotide in the promoter region, resulting in gene silencing and the function loss of some tumor suppressor genes such as *MLH1, APC, MCC, MGMT*, and several others [44]. As the CIMP refers to the presence of multiple hypermutated genes, Weisenberger et al. have proposed a panel of five markers, which are CACNA1G, IGF2, NEUROG, RUNX3, and SOCS1. CIMP+ was defined by the methylation of 3 to 5 markers and CIMP- by the hypermethylation of 0 to 2 loci [45]. Other studies classified tumors to CIMP-high (CIMP-H) and CIMP-low (CIMP-L) or CIMP-0 [46].

Clinically, CIMP-H CRCs have been associated with female sex, older age, right-sided tumor location, and advanced stage. At the pathologic level, CIMP-H tumors showed higher rates of tumor-infiltrating lymphocytes, Crohn-like infiltrates, perineural, lymphovascular invasion, and higher levels of Fusobacterium nucleatum. With regard to molecular characteristics, this tumor subtype was shown to exhibit a higher prevalence of *BRAF* and *PIK3CA* mutation (OR: 20.17 (95% CI: 13.54–30.05); 1.61 (95% CI: 1.24–2.10), respectively) and more likely to have higher MSI status (OR: 10.95 (95% CI: 8.49–14.13)). Additionally, there was an inverse association of CIMP-H tumors with *TP53* and *KRAS* mutations, and no association has been reported with *APC* mutation [42].

Why CIMP is consistently associated with *BRAF* mutations has long been a debated question. In 2019, Tao et al. provided compelling evidence that solved this long-standing question suggesting that through the aging-like acquisition of DNA methylation, BRAF mutated cells may survive by suppressing senescence and activating stem cell pathways [47].

## 3. Molecular Classification of CRCs and Associated Features

### 3.1. Molecular Subtypes of CRC

As we reviewed earlier, there are three major mechanisms of genetic instability in CRC: CIN, MSI, and CIMP. Many studies have tried to establish a molecular classification for CRCs, but these did not lead to a single systematic classification [48].

A systematic molecular pathological classification has been proposed by The Cancer Genome Atlas (TCGA) in 2012 [49] and another one by the Consensus Molecular Subtype (CMS) Consortium in 2015 [50].

Despite the heterogeneity of CRCs, the ancient classification by TCGA has divided CRCs into two subtypes, which are characterized by a specific morphology and molecular alteration.

- The hypermutated cancers, representing 16% of CRCs. Three-quarters of this group have a high MSI as a result of dMMR, and the other one-quarter corresponds to ultramutated cancers with polymerase-*ε(POLE)* mutations.
- The nonhypermutated cancers, which account for 84% of CRC cases. The tumors of this group are MSS, which harbors a higher frequency of alterations in somatic DNA and common mutations in *APC*, *TP53, KRAS, SMAD4*, and *PIK3CA* genes [48, 49].

**Figure 2:**
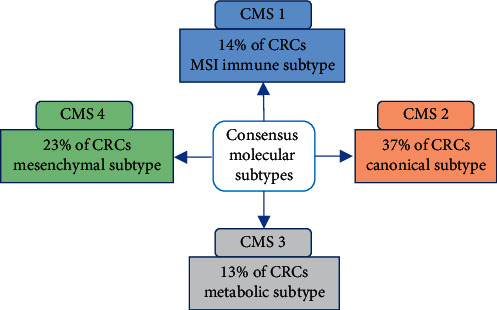
Consensus molecular subtypes classification of colorectal cancer. CMS: Consensus Molecular Subtype; CRCs: colorectal cancers; MSI: microsatellite instability.

In 2015, an international consortium has analyzed a large-scale data sharing, aiming to establish a new and universal molecular classification and facilitate its clinical implication. The panel experts evaluated six CRC subtyping algorithms from six studies [51–56] and also the data of TCGA, to develop a novel classification of four CMS groups (Figure 2) [50].

- CMS1 (microsatellite instability immune, 14%): Almost all patients with MSI were regrouped in this group, characterized by hypermutated profile, especially in *MLH1* gene, and high level of *BRAF* mutations. CMS1 patients have a strong immune activation, reflected by a high level in gene expression, associated with a diffuse immune infiltrate and upregulation in immune response pathways (PD1 activation, NK cells, Th1 cells, and cytotoxic T-cell infiltration signatures) [50]. This immune activation is a new feature of MSI CRCs [57]
- CMS2 (canonical, 37%): It includes patients with higher CIN, which have a high level of somatic copy number alterations (SCNAs). Conversely to CMS1, CMS2 showed a strong upregulation of WNT and MYC downstream targets and epithelial differentiation. Compared with other groups, CMS2 exhibits more frequently copy number gain in oncogenes and copy number losses in tumor suppressor genes [50]
- CMS3 (metabolic,13%): It is characterized by dysregulation of many metabolic pathways (glucose pentose, nitrogen, fatty acid, etc.), CIN with fewer SCANs, higher prevalence of CIMP-low, and higher *KRAS* mutations compared with other groups. Almost 30% of CMS3 was hypermutated, which results in more MSI samples compared with CMS2 and CMS4 [50]
- CMS4 (mesenchymal, 23%): Higher CIN and increased level of SCNAs, with strong expression of epithelial-to-mesenchymal transition (EMT) genes and activation of TGF-*β* signaling. CMS4 overexpresses more commonly proteins that are implicated in stromal infiltration and angiogenesis and exhibits higher expression level of mesenchymal protein pathways [50]

Finally, there were tumors with mixed features (13%) that possibly represent either a transition phenotype or intratumoral heterogeneity [50].

### 3.2. Clinical and Prognostic Associations of the CMSs

CMS1 has been found to be significantly more common in females with higher histological grades and right-sided lesions, conversely to CMS2 tumors which are more frequently left-sided. CMS4 is diagnosed often at advanced stages compared with other subtypes [50].

By analyzing data from patients enrolled in the PETACC-3 clinical trial [58], Guinney et al. [50] concluded that CMS can be used as a prognostic factor [59], and this was also supported by other studies [60–65]. However, as no targeted therapy regimens are available for primary CRC, stratifying tumors using CMS as a prognostic tool needs further evaluation [66]. In a recent large monocenter cohort study including 308 CRC tumors, Purcell et al. concluded that although CMS alone does not surpass TNM staging in terms of prognostication, their combination seems of interest [66]. CMS3 tumors showed lower OS of stage 2 CRCs than other subtypes, whereas stage 2 patients with a good prognosis exhibited immune activation and upregulation of tumor suppressor genes [66].

After analyzing data from the PETACC-3 clinical trial, CMS4 is reported to have the worst OS and relapse-free survival (RFS) in response to fluorouracil (FU)/leucovorin (LV) and irinotecan adjuvant regimen. CMS1 tumors have also a very poor survival rate after relapse, conversely to CMS2 that tends to have superior survival rates after relapse [50].

An *in vitro* study by Sveen et al. reported a strong response to EGFR and human epidermal growth factor receptor 2 (HER2) inhibitors for the CMS2 group, while CMS1 and CMS4 demonstrated higher sensitivity to HSP90 inhibitors [67]. CMS1 has shown worse PFS and OS in response to anti-EGFR therapy, whereas CMS2 showed particularly better PFS and OS compared with other groups [68]. This was supported by analyzing data from CALGB/SWOG 80405 phase III trial, which found more OS benefit after anti-VEGF than anti-EGFR treatments for the CMS1 group (*P* < 0.001) [60]. However, patients in the CMS2 group treated with cetuximab were found to have better OS compared to those treated with bevacizumab (*P*=0.0046) [60]. Additionally, analysis of data from the FIRE-3 trial showed significantly better OS for CMS4 group after cetuximab plus FOLFIRI treatment compared to bevacizumab plus FOLFIRI in wild-type RAS mCRC [61].

## 4. Targeted Therapy in Metastatic CRC

5-fluorouracil (5-FU) was the first chemotherapy regimen used for the treatment of advanced CRC. After many failed combination regimens to improve its response rate, leucovorin has shown for the first time an advantage over 5-FU alone in terms of tumor response rate in 1992 [69]. Thereafter and over the past two decades, other drugs have shown more improvement in terms of survival, either with 5-FU or alone, such as irinotecan, capecitabine, and oxaliplatin [70]. With the understanding of molecular pathways in CRC, a number of targeted biologic therapies have been approved by the FDA. The first ones were the monoclonal antibodies (mAbs) targeting VEGF (bevacizumab) and the EGFR (cetuximab and panitumumab). The advancement in chemotherapy and targeted drugs has led to significantly improve overall survival to over 40 months for mCRC patients [8].

### 4.1. Antiangiogenic Inhibitors Targeting VEGF or Its Receptors

Angiogenesis is a mechanism that allows the creation of new blood vessels from preexisting ones to supply cancerous cells. It plays a crucial role in tumor initiation, growth, and metastasis [71]. For a long time, targeting the angiogenic pathway has been considered an important approach for cancer therapy. Although more than 40 molecules have been found to play an important role in blood vessel recruitment, most studies have focused on VEGF and its receptors [70]. VEGF signaling pathway is a key contributor in the process of angiogenesis, and high levels of VEGF ligands and its receptors activity were shown to be related to poor prognosis in CRC and other cancers [72–74].

Bevacizumab is the first anti-VEGF drug that has been approved by the FDA in 2004 for the treatment of patients with mCRC, initiating its use as standard first-line treatment in combination with chemotherapy. Bevacizumab is a humanized monoclonal antibody that binds to VEGF-A, preventing its binding on its receptors. According to a phase II clinical trial of Kabbinavar et al., bevacizumab with FU and LV, in the first line of mCRC treatment, was found to improve significantly the progression-free survival (PFS) and response rate (RR) compared to FU/LV plus placebo (PFS: 9 vs. 5.2 months; hazard ratio (HR), 0.005; *P* < 0.001; RR: 40% vs. 17%; *P*=0.029) but did not improve the median OS (21.5 vs. 13.8 months; *P*=0.137). The optimal dose of bevacizumab was 5 mg/kg [75]. In a phase III trial, significant improvement in OS was demonstrated with irinotecan, FU and LV (IFL), and bevacizumab compared to IFL plus placebo (20.3 vs. 15.6 months; HR, 0.66; *P* < 0.001) [76]. Other clinical trials have tested new combinations with bevacizumab in mCRC and showed a significant improvement in OS or PFS in first-line setting such as LV calcium, FU, and oxaliplatin-4/capecitabine and oxaliplatin (FOLFOX-4/Xelox), FU, LV, and irinotecan (FOLFIRI) and capecitabine [77–79]. Bevacizumab with FOLFIRI showed a better PFS and OS compared to bolus FU/LV with irinotecan (mIFL) and oral capecitabine with irinotecan (capeIRI) [80]. In the second-line setting, bevacizumab was tested with FOLFOX-4 and was shown to improve the median PFS and the RR compared to FOLFOX-4 alone (PFS: 7.3 vs. 4.7 months; HR, 0.61; *P* ≤ 0.0001; RR: 22.7% vs. 8.6%; *P* ≤ 0.0001) [81]. The ML18147 phase III clinical trial concluded that the continuation of bevacizumab with switching second-line-based chemotherapy improved significantly OS compared to chemotherapy alone or bevacizumab alone [82]. Based on the positive results of these studies, the use of bevacizumab with fluoropyrimidine-oxaliplatin or fluoropyrimidine-irinotecan chemotherapy has been approved by the FDA as a second line for mCRC in 2013.

After bevacizumab, three other antiangiogenic agents have been approved for mCRC: aflibercept, regorafenib, and ramucirumab.

Aflibercept (also known as VEGF Trap, AVE0005) is a recombinant fusion protein that acts as a receptor, binding to human VEGF-A, VEGF-B, and placental growth factor (PGF). It is composed of extracellular domains of human VEGF receptors 1 and 2, fused to the Fc portion of human immunoglobulin (Ig)G1. Aflibercept has a high-affinity ligand trap and prevents these ligands to bind to their endogenous receptors [83]. VELOUR phase III trial showed that treatment with FOLFIRI and aflibercept conferred a significant benefit in terms of OS and PFS compared with FOLFIRI and placebo (OS: 13.5 vs. 12.1 months; HR, 0.82; *P*=0.0032; PFS: 6.9 vs. 4.7 months; HR, 0.76; *P* < 0.001) [84]. Based on the positive results of this clinical trial, aflibercept has been approved after oxaliplatin failure as a second-line treatment in combination with FOLFIRI in mCRC patients by the FDA in August 2012 and by the European Medicines Agency (EMA) in February 2013.

Regorafenib is an oral multikinase inhibitor that inhibits angiogenic tyrosine kinases (vascular endothelial growth factor receptor 1 to 3 (VEGFR1-3), platelet-derived growth factor receptor (PDGFR- *β*), and fibroblast growth factor receptor (FGFR1)). It also blocks BRAF and oncogenic receptor tyrosine kinases (RTKs), such as RET and KIT [85]. The CORRECT phase III trial showed that treatment with regorafenib conferred a significant improvement in OS compared with the placebo arm, for mCRC that was refractory to standard therapy (6.4 vs. 5 months; HR, 0.77; *P*=0.0052) [86]. Based on this trial, regorafenib has been approved by the FDA in September 2012 for the treatment of mCRC patients who have been treated previously with fluoropyrimidine-, oxaliplatin- and irinotecan-based chemotherapy, an anti-VEGF therapy, and an anti-EGFR therapy if KRAS wild type.

Ramucirumab is a monoclonal human antibody that has a high affinity to VEGFR-2, the essential receptor of the VEGF angiogenic signaling pathway. The RAISE phase III clinical trial has found that ramucirumab plus FOLFIRI as second-line treatment of mCRC improved significantly OS compared with FOLFIRI plus placebo (13.1 vs. 11.7 months, HR, 0.84; *P*=0.0219) [87]. Based on these results, ramucirumab (Cyramza) in combination with FOLFIRI has been approved by the FDA as a second-line option for mCRC on April 24, 2015.

Other small multitargeted receptor tyrosine kinase inhibitors (RTKIs) are under investigation for mCRC treatment, such as Famitinib which targets VEGFR2, PDGFR, and c-Kit [88], as well as Fruquintinib and Cediranib which inhibit VEGF1-3 receptors [89, 90]. The main antiangiogenic RTKIs agents, which are under clinical investigation in mCRC, were summarized in Table 1.

### 4.2. Anti-EGFR Inhibitors

Epidermal growth factor receptor (EGFR/ERBB1) is a member of the erythroblastosis oncogene B (ERBB) family, which also consists of three other receptors, HER2 (ERBB2), HER3 (ERBB3), and HER4 (ERBB4) [91]. Ligands such as EGF, TGF*α*, Amphiregulin (AREG), and Epiregulin (EREG) activate EGFR by binding to its extracellular domain, leading to the activation of the tyrosine kinase domain in the cytoplasm, which stimulates two major signal-transduction pathways, RAS/RAF/MEK/ERK (mitogen-activated protein kinase (MAPK)) pathway and PI3K/AKT pathway. These two intracellular pathways play key roles in cell proliferation, survival, and migration [92, 93].

There is great evidence that ERBB family members have an important role in the initiation and survival of several solid cancers. Over the last years, many studies have shown the importance of constitutive activation of the EGFR pathway in cancer cell proliferation, stopping apoptosis, and activating metastasis [94–96]. This activation may occur through receptor overexpression and ligand-dependent and independent mechanisms [93]. After the research of Masui et al. in 1983 that provided evidence on the activity of anti-EGFR drugs against epidermoid carcinoma cell growth *in vivo* [97], many studies and clinical trials focused on two classes of anti-EFGR agents, which are the anti-EGFR monoclonal antibodies (cetuximab and panitumumab) and the small-molecule EGFR tyrosine kinase inhibitors [92].

Cetuximab is an anti-EGFR monoclonal antibody mAb (recombinant immunoglobulin G1 (IgG1)), which has been approved by the FDA in 2004, for the treatment of mCRC in combination with irinotecan after irinotecan-based refractory chemotherapy, or as a single agent for mCRC patients who are intolerant to irinotecan. This approval was based essentially on an open-label, randomized trial that showed a significant improvement in terms of RR and PFS for cetuximab with irinotecan, compared to cetuximab alone, in patients who were refractory to irinotecan-based chemotherapy (RR: 22.9 vs. 10.8%; *P* : 0.007; PFS: 4.1 vs. 1.5; *P* < 0.001) [98]. The CRYSTAL phase III clinical trial showed that cetuximab with FOLFIRI as a first line of treatment increased the RR by 10% (adjusted odds ratio (OR): 1.40; *P*=0.004) and reduced the risk of mCRC tumor progression by 15% (HR, 0.85; *P*=0.048) compared to FOLFIRI alone, and this benefit has been limited to KRAS wild-type groups (RR: 59.3% vs. 43.2% (OR, 1.9); PFS: 9.9 vs. 8.2; HR, 0.68; *P*=0.02). No improvement in OS has been reported in this study, similarly to the previously mentioned trial [99]. Consequently, cetuximab in combination with FOLFIRI has been approved by the FDA for KRAS wild-type mCRC patients as a first-line treatment. FOLFOX4 has been tested in phase II of OPUS clinical trial and showed an improvement in overall response rate (ORR) and a lower risk of disease progression in patients with KRAS wild-type disease treated with cetuximab, as compared to those who received FOLFOX4 alone (ORR = 61 vs. 37%; OR: 2.54; *P*=0.011; PFS: 7.7 vs. 7.2 months; HR, 0.57; *P*=0.0163, respectively) [100]. A pooled analysis of the CRYSTAL and OPUS randomized clinical trials has shown that combining cetuximab to chemotherapy (FOLFIRI or FOLFOX4) in patients with KRAS wild-type disease improved significantly OS (HR, 0.81; *P*=0.0062), PFS (HR, 0.66; *P* < 0.0001), and ORR (OR, 2.16; *P* < 0.0001), compared with chemotherapy alone. In contrast, BRAF mutation status did not show any significant difference in response to cetuximab, but it was a negative prognostic biomarker [101].

Recently, on April 8, 2020, the FDA approved encorafenib in combination with cetuximab for the treatment of mCRC with a BRAF V600E mutation, after prior therapy. This approval was based on the phase III BEACON CRC study, which showed an improvement of median OS for mCRC patients with BRAF V600E treated with cetuximab and encorafenib compared to cetuximab alone (8.4 vs. 5.4 months; HR, 0.6; *P* < 0.001) [102]. In the second-line setting, the phase III EPIC study has shown that cetuximab and irinotecan improved significantly PFS and RR and resulted in a better quality of life, after oxaliplatin and fluoropyrimidine failure, compared with irinotecan alone [103]. Additionally, cetuximab alone was shown to improve OS and PFS, in wild-type KRAS patients who failed all other treatments, compared with best supportive care (BSC) alone (9.5 vs. 4.8 months; HR, 0.55; *P* < 0.001 and 3.7 vs. 1.9 months; HR, 0.40; *P* < 0.001, respectively) [104].

Panitumumab is also an anti-EGFR monoclonal antibody. Conversely to cetuximab, it is a fully human IgG2, which has shown reduced immunogenic reactions and high affinity and specificity for the EGF receptors [105]. In phase III clinical trial, panitumumab and BSC showed a significant improvement in PFS for mCRC patients who had progressed after standard chemotherapy, compared with those receiving BSC alone [106]. PRIME phase III study has shown that panitumumab with FOLFOX4 as the first line of treatment for patients with wild-type KRAS mCRC improves significantly PFS compared with FOLFOX4 alone (10 vs. 8.6 months; HR, 0.8; *P*=0.01) [107]. As a second-line treatment, panitumumab with FOLFIRI has shown significant improvement in terms of PFS, compared with FOLFIRI alone for mCRC patients without KRAS mutations (5.9 vs. 3.9 months; HR, 0.71; *P*=0.004) [108]. In 2006, the FDA approved panitumumab as a single agent for the treatment of mCRC KRAS wild-type after chemotherapy regimens failure, and in 2014, it was approved in combination with FOLFOX for patients with wild-type KRAS mCRC in the first-line setting.

## 5. Resistance to Anti-EGFR Therapy

Generally, resistance to targeted drugs englobes primary (de NOVO or innate) and secondary resistance (acquired resistance) [109]. Patients with primary resistance exhibit gene mutations, allelic loss, or gene overexpression, which inactivate or reduce the effectiveness of the drug targets. As an example, *RAS, BRAF,* and *PIK3CA* mutations and loss of PTEN and HER2 overexpression have been involved in the primary resistance to anti-EGFR therapy [110]. It has been shown that 40% of all mCRC patients will derive benefit from these agents [111]. Hence, identifying predictive biomarkers of response to anti-EGFR mAbs is of utmost importance. With regard to secondary resistance, the underlying mechanisms are the acquired EGFR (S492R) mutation, genetic alterations of RAS, BRAF, HER2, and MET, and the selection effect of preexisting subclones that confer primary resistance to anti-EGFR mAbs. These mechanisms and others have been reviewed by Misale et al. and Zhoa et al. [112, 113].

### 5.1. Primary Resistance

RAS mutations: the frequency of *KRAS* gene mutations is estimated at 40% in all CRCs. These mutations were shown to directly activate the MAP kinase signaling pathway, leading to anti-EGFR mAbs resistance [70]. Mutations located on exon2 (codon 12 or 13) were considered the first and the most important predictive biomarker for the nonresponse to anti-EGFR mAbs. These alterations represent 85–90% of *KRAS* mutations in CRC, and approximately 40% of mCRC patients were found to be mutation carriers [113, 114]. Based on the results of many studies that reported a nonbenefit from anti-EGFR mAbs and shorter PFS, OS, and RR for patients with *KRAS* exon2 mutations compared with wild-type patients [104, 115–117], the use of anti-EGFR mAbs has been limited by the FDA in 2009 to KRAS exon 2 wild-type mCRC patients [118]. Moreover, other *KRAS* mutations in exon 3 (codons 59 and 61) and exon 4 (codons 117 and 146) and mutations of the *NRAS* isoform (exons 2, 3, and 4) were identified in 15–20% of *KRAS* exon 2 wild-type patients and were found to be related to low PFS and OS in patients treated with cetuximab and panitumumab [119–122]. Of note, not all patients with wild-type RAS respond to anti-EGFR treatment; that is why research and identification of other biomarkers are of utmost importance.

BRAF mutations: BRAF is a downstream effector, which is directly regulated by RAS. Mutations in the *BRAF* gene represent 5–9% of CRCs and may lead to direct activation of the RAS/RAF/ERK pathway [123]. V600E is considered the most commonly reported mutation in BRAF, accounting for more than 95% of all identified mutations. This mutation causes direct activation of the MAP kinase pathway, leading to resistance to anti-EGFR mAbs [124]. Many studies reported that BRAF V600E has been associated with poorer PFS and OS in patients treated with anti-EGFR mAbs [125, 126]. However, a meta-analysis of eight randomized controlled trials by Rowland et al. concluded that there is insufficient evidence to consider BRAF as a predictive biomarker of benefit from anti-EGFR mAbs therapy for RAS wild-type mCRC, as there was no statistically significant difference in OS and PFS between RAS wild-type/BRAF wild-type and RAS wild-type/BRAF mutant [127].

PIK3CA mutations and PTEN loss: PI3K-AKT-mTOR signaling pathway is also known to be activated by EGFR, leading to cell proliferation, cell growth, and apoptosis suppression in CRCs [128]. Mutations on *PIK3CA*, which represent 10–18% of CRCs, can lead to direct activation of the PI3K/AKT pathway and cause resistance to anti-EGFR mAbs [113]. Mutations in exons 9 and 20 account for 80% of all *PIK3CA* mutations and result in its activation and the activation of its downstream signaling pathway [129]. A study by Sartore-Bianchi et al. including 110 mCRC-treated patients reported that *PIK3CA* mutations conferred significant clinical resistance to cetuximab and panitumumab [130]. Another large retrospective consortium analysis study showed that only *PIK3CA* exon 20 mutations have been associated with lower RR, PFS, and OS as a response to cetuximab plus chemotherapy compared with wild types, whereas exon 9 mutations showed no effect [131]. A meta-analysis by Mao et al., which included 576 mCRC patients, found that the objective response rate to anti-EGFR mAbs in the KRAS wild-type group was lower in patients with *PIK3CA* exon 20 mutations, but this difference was not significant due to the limited sample size. However, this study suggested that *PIK3CA* exon 20 mutations may predict the resistance to anti-EGFR mAbs in KRAS wild-type mCRC patients [132].

*PTEN* is considered a suppressor gene, due to its role in the negative regulation of AKT. Therefore, loss of *PTEN* expression or function leads to persistent activation of the PI3K-AKT-mTOR signaling pathway, which results in permanent cell proliferation and growth [133]. Some studies have found that *PTEN* can be a useful predictive biomarker for the response to anti-EGFR mAbs therapy, particularly in the KRAS wild-type [134–137]. In contrast, other studies did not find a significant difference in response to anti-EGFR therapy between PTEN-positive and PTEN-negative groups [138–140]. To confirm the role of PTEN in anti-EGFR resistance, other large clinical studies are warranted.

Level of EGFR ligand expression: in addition to studying the downstream EGFR signaling pathway in anti-EGFR resistance, the upregulation has also been investigated in some studies, especially in an intact downstream EGFR pathway [141–144]. In an exploratory cetuximab monotherapy clinical trial, cetuximab efficacy has been found to be related to KRAS wild-type and high gene expression levels of AREG and EREG in mCRC patients [145], whereas other clinical trials confirmed that AREG and EREG expression levels have predictive power to anti-EGFR therapy only for KRAS wild-type patients [142, 146]. A recent study that analyzed tumor tissue of 688 patients participating in FIRE-1, CIOX, and FIRE-3 clinical trials has confirmed that AREG high level is a positive prognostic biomarker for anti-EGFR therapy in mCRC. AREG high level was significantly associated with high OS and PFS compared to a low level (26.2 vs. 21.5 months; *P*=0.007) (10.0 vs. 8.1 months; *P*=0.001), respectively [141]. EGFR ligand's high expression level can be related to tumors' dependence on the EGFR signaling pathway, which explains its predictive power in mCRC, but it can also occur as a consequence of epigenetic regulation [147].

STAT3: STAT3 is a transcription factor belonging to the STAT family. It is an essential component of the JAK/STAT signaling pathway, which is activated by EGFR and other receptors [148]. Phosphorylated by JAK, STAT3 plays an important role in cell proliferation, survival, and apoptosis activities [113, 149]. Persistent STAT3 activation may have a key role in anti-EGFR therapy resistance in mCRC. A retrospective study by Dobi et al. reported a significant improvement in time to progression (TTP) and OS for negative phospho-STAT3 compared to positive phospho-STAT3 group (TTP: 6.3 vs. 5.4 months; *P* < 0.01; OS: 13.1 vs. 9.4 months; *P*=0.02), among 94 mCRC patients who were treated with cetuximab and chemotherapy in the second-line setting or beyond [150]. Another study by Ung et al. indicated a key role of STAT3 in promoting resistance to anti-EGFR treatment and showed that STAT3 activity can be inhibited by the anti-EGFR inhibitors in wild-type K-RAS colon cancer cell lines, suggesting that anti-EGFR therapy combined with STAT3 inhibitors may provide a therapeutic benefit for mCRC patients [151].

### 5.2. Secondary Resistance

Acquisition of KRAS mutation: as we mentioned previously, the RAS/RAF signaling pathway plays an important role in primary resistance to anti-EGFR mAbs in mCRC, but it has also been involved in acquired resistance [152]. A report by Diaz et al. found that 38% of KRAS wild-type patients who received panitumumab had KRAS mutations in their sera, occurring after five or six months of the treatment [153]. Interestingly, a mathematical model from this study showed that resistance mutations in KRAS were present in a clonal subpopulation within the tumors before initiating panitumumab therapy [153]. Another study reported that 55% (6/11) of patients who developed cetuximab or panitumumab resistance harbored secondary k-RAS mutations, and 9% (1/11) had K-RAS amplification [154]. KRAS variants were detectable in plasma of cetuximab-treated patients 10 months before radiographic documentation of disease progression [154].

EGFR S492R mutation: Montagut et al. were the first to report in 2012 that EGFR S492R mutation confers acquired resistance in mCRC patients treated with cetuximab, but not in those who were treated with panitumumab. In their study, 20% of patients who showed resistance to cetuximab harbored EGFR S492R mutation [155]. Later, a larger cohort study including 505 mCRC KRAS exon 2 wild-type patients suggested that EGFR S492R mutation was not involved in primary resistance to cetuximab [156]. In the ASPECCT study, a randomized controlled phase III trial, EGFR S492R mutation was detected, after analyzing liquid biopsies, in 16% of patients treated with cetuximab compared to 1% in those receiving panitumumab [157].

Amplification of HER2: HER2 can activate the RAS/RAF/ERK and PI3K/AKT pathways through its heterodimerization with EGFR and HER3, which are all members of the ERBB receptors tyrosine kinase family. For this reason, HER2 is considered a potential biomarker for the sensitivity to anti-EGFR therapy [91]. Studies using patient-derived xenografts showed that HER2 amplification or the overexpression of heregulin ligand leads to cetuximab resistance especially in KRAS and BRAF wild-type cancers [158, 159]. These findings have been supported by clinical data from a large retrospective cohort study by Martin et al. showing that HER2 gene copy number may confer resistance to anti-EGFR therapy in KRAS wild mCRC type [160]. HER2 amplification may also be involved in primary resistance, but considering its low frequency (2% of mCRC patients), it is mainly considered a mechanism of secondary resistance to anti-EGFR [113].

MET amplification: mesenchymal-epithelial transition factor (c-MET) is a tyrosine kinase receptor, which is encoded by the protooncogene *MET*. Binding to its ligand the hepatocyte growth factor (HGF), c-MET induces cell proliferation, growth, survival, and angiogenesis, through the activation of PI3K/AKT, RAS/RAF/ERK, STAT3, and nuclear factor-*κ*B (NF-*κ*B) signaling pathways [161]. Liska et al. have confirmed the importance of MET activation in restoring the MAPK and AKT pathways during anti-EGFR therapy in CRC cell lines [162]. In both *in vitro* and *in vivo* settings, Bardelli et al. demonstrated that MET amplification confers acquired resistance in patients who were KRAS wild-type during anti-EGFR therapy. Importantly, findings from this study supported using blood tests to monitor the emergence of MET amplification in patients undergoing anti-EGFR therapies, as the amplification of the MET locus has been present in circulating tumor DNA before any clinical evidence of relapse [163]. Another study by Troiani et al. showed that TGF-*α* overexpression induces the EGFR-MET interaction leading to subsequent MET pathway activation and MET acquired resistance and suggested that the inhibition of MET expression restores the sensitivity to cetuximab in CRC cell lines [164]. Nonetheless, MET amplification occurs only in 1% of unselected mCRC patients [163, 165], making it a weak predictive biomarker of primary resistance to anti-EGFR therapy in mCRC [113].

The subclone selection: the notion of secondary resistance not only consists of the development of new mutations during the therapy but can also include the selection of a low-frequency subclone that conferred primary resistance under the target therapy pressure [166]. To determine whether the acquired resistance to cetuximab in mCRC patients is due to novel mutations or the selection of preexisting subclones, Misale et al. compared gene copy number and the mutational profile of parental and resistant cell lines [154]. This study found that KRAS G13D mutation and KRAS amplification were present in parental cells with low frequency, which supports the theory of subclonal selection [154]. However, other mutations including KRAS G12R and EGFR S492R have been identified only in resistant cells [154, 156], which corroborates that some mutations may occur only during the EGFR mAbs treatment.

### 5.3. Overcoming Resistance to Anti-EGFR Therapy

In order to overcome resistance to cetuximab/panitumumab in mCRC patients, research studies have been focusing on widening therapeutic choices by testing new monoclonal antibodies against EGFR receptors (Duligotuzumab, Futuximab, etc.), as well as those against HER2, HER3, and IGF-1R receptors (Table 2). Using tyrosine kinase inhibitors to target the EGFR receptor and its downstream pathway is another promising active research area (Table 3).

## 6. Conclusion

The advancements in molecular technologies in recent decades, especially sequencing techniques, have led to a better understanding of the genomic landscape of CRC and hence increased dramatically our knowledge about the carcinogenesis process. This unprecedented information has been directly harnessed to establish molecular classifications that provide deeper insights into the biology of CRC. Available evidence suggests that the CMS classification might have a predictive and prognostic value, and also it can help to guide drug development and application.

The molecular understanding of CRC carcinogenesis has also led to developing targeted drugs such as anti-VEGF mAbs, anti-EGFR mAbs, and multikinase inhibitors, which improved the survival rates of mCRC patients. However, emerging primary or secondary resistance to current targeted therapies, especially to anti-EGFR mAbs, remains a major problem in clinical practice. Understanding resistance mechanisms, identifying new biomarkers and other targetable pathways is of paramount importance to optimize therapeutic choices and improve survival for resistant patients.

## Figures and Tables

**Table 1:** Antiangiogenic RTKIs agents under clinical trials investigation in mCRC.

Drugs	Target	Number of participants	Setting	Treatment	Primary outcome measures	Phase	Identifiers
Vatalanib (PTK787/ZK 222584)	VEGFR1-3, FGFR1-3, PDGFR*α*-*β*	1168	1st line mCRC	Oxaliplatin/5FU/Leucovorin ± vatalanib	PFS	III	NCT00056459
		855	2nd line mCRC		OS		NCT00056446
Nintedanib (BIBF1120)	VEGFR1-3, FGFR1-3, PDGFR*α*-*β*	768	Refractory mCRC	BSC ± Nintedanib	PFS/OS	III	NCT02149108
		54	Refractory mCRC	mFOLFOX6 ± Nintedanib	PFS	III	NCT01362361
Semaxanib (SU5416)	VEGFR2	710	1st line mCRC	Leucovorin and Fluorouracil ± Semaxanib	OS	III	NCT00004252
Brivanib	VEGFR2/FGFR1	750	mCRC kRAS wild-type (refractory)	Cetuximab ± Brivanib	OS	III	NCT00640471
Sunitinib	PDGFR-*β*, VEGFR2,	768	1st line mCRC	FOLFIRI ± Sunitinib	PFS	III	NCT00457691
Fruquintinib	VEGFR1-3	416	3rd line mCRC	Fruquintinib vs. placebo	OS	III	NCT02314819
Cediranib (AZD2171)	VEGFR1-3	1814	1st line mCRC	Beva + folfox vs. beva + Cediranib	PFS	II/III	NCT00384176
Sorafenib	VEGFR1-3 PDGFR-*β* BRAF	101	2nd line mCRC	FOLFOX6/FOLFIRI ± Sorafenib	PFS	II	NCT00889343
Vanucizumab	VEGF-A Angiopoietin-2	197	1st line mCRC	mFOLFOX6 + beva vs. mFOLFOX6 + Vancizumab	PFS	II	NCT02141295
Famitinib	VEGFR2-3, c-Kit, PDGFR	154	3rd line advanced mCRC	Famitinib vs. placebo	PFS	II	NCT01762293
Axitinib	VEGFR1-3	70	1st line maintenance therapy	Axitinib alone after FOLFOX/beva in 1st line	PFS	II	NCT01490866
Apatinib	VEGFR2	54	Refractory mCRC progressed after 2nd line	Apatinib alone	PFS	II	NCT03190616

mCRC: metastatic colorectal cancer; 5FU: 5-fluorouracil; FOLFOX: oxaliplatin in combination with 5-fluorouracil and folinic acid; FOLFIRI: irinotecan in combination with 5-fluorouracil and folinic acid; BSC: best supportive care; Beva: bevacizumab; PFS: progression-free survival; OS: overall survival; VEGFR: vascular endothelial growth factor receptor; FGFR: fibroblast growth factor receptor; PDGFR: platelet-derived growth factor receptor.

**Table 2:** mAbs targeting EGFR pathway under clinical investigation.

Drugs	Target	Setting	Treatment	Phase	Identifiers
Trastuzumab	HER2	HER2-positive wild KRAS mCRC	Trastuzumab + Lapatinib or Pertuzumab	II	NCT03225937
			Trastuzumab + Tucatinib		NCT03043313
Pertuzumab	HER2	2nd line of advanced or mCRC	Pertuzumab + cetuximab	I/II	NCT00551421
MEHD7945A (Duligotuzumab)	EGFR/HER3	2nd line K-Ras wild-type mCRC	MEHD7945A + FOLFIRI vs. Cetuximab + FOLFIRI	II	NCT01652482
SYM004	EGFR	mCRC K-Ras WT acquired resistance to Anti-EGFR mAbs	SYM004 vs. BSC	II	NCT02083653
CPGJ 602	EGFR	2nd line mCRC KRAS WT	CPGJ 602 vs. Cetuximab	I	NCT03356158
Futuximab	EGFR	Chemotherapy-refractory mCRC	Futuximab vs. SYM004	II	NCT03549338
SCT 200	EGFR	Wild-type RAS and RAF mCRC	SCT200	II	NCT03405272
Dalotuzumab (MK-0646)	IGF-1R	Wild-type KRAS mCRC	Dalotuzumab + Cetuximab + irinotecan	II/III	NCT00614393
Ganitumab (AMG-479)	IGF-1R	Mutant KRAS mCRC	AMG-479 + FOLFIRI vs. FOLFIRI alone	II	NCT00813605
Cixutumumab (IMC-A12)	IGF-1R	2nd line mCRC kRAS wild-type	Irinotecan and Cetuximab ± IMC-A12	II	NCT00845039

mAbs: monoclonal antibodies; mCRC: metastatic colorectal cancer; FOLFIRI: irinotecan in combination with 5-fluorouracil and folinic acid; BSC: best supportive care; HER: human epidermal growth factor receptor; EGFR: epidermal growth factor receptor; IGF-1R: insulin-like growth factor 1 receptor; KRAS: Kirsten rat sarcoma viral oncogene.

**Table 3:** TKIs targeting EGFR pathway under clinical investigation.

Drugs	Target	Setting	Treatment	Phase	Identifiers
Erlotinib	EGFR	2nd line k-RAS WT mCRC	Erlotinib + panitumumab ± irinotecan	II	NCT00940316
Neratinib	EGFR/HER2	KRAS/NRAS/BRAF/PIK3CA wild-type mCRC	Neratinib + Trastuzumb vs. Neratinib + Cetuximab	II	NCT03457896
Sapitinib (AZD8931)	EGFR/HER2/3	Recurrent or metastatic CRC	AZD8931 + FOLFIRI	II	NCT01862003
Tucatinib	HER2	HER2 positive CRC	Tucatinib + trastuzumab	II	NCT03043313
Lapatinib (GSK572016)	EGFR/HER2/erk-1/2	2nd line advanced or mCRC	Lapatinib + capecitabine	II	NCT00574171
Vemurafenib	BRAF (V600E)	BRAF V600E mutation and advanced CRC	FOLFIRI + Cetuximab + Vemurafenib	II	NCT03727763
Dabrafenib	BRAF (V600E)	BRAF V600E mutation mCRC	Dabrafenib + trametinib + spartalizumab	II	NCT03668431
Encorafenib (LGX-818)	BRAF (V600E)	MSS/BRAF V600E mCRC	Encorafenib + Cetuximab + nivolumab	I/II	NCT04017650
BMS-908662	BRAF	Mutant BRAF mCRC	BMS-908662 + cetuximab	I/II	NCT01086267
Binimetinib (MEK162)	MEK1/2	RAS positive mCRC	Binimetinib + mFOLFIRI	I	NCT02613650
Cobimetinib	MEK1	Locally advanced and metastatic CRC	Cobimetinib + Atezolizumab vs. Regorafenib	III	NCT02788279
Trametinib	MEK1/2	RAS/RAF mutant and TP53 WT mCRC	Trametinib + HDM201	I	NCT03714958
Selumetinib (AZD6244)	MEK1/2	2nd line k-RAS BRAF mCRC	Selumetinib + irinotecan	II	NCT01116271
Alpelisib (BYL719)	PI3K	BRAF mutant mCRC	Alpelisib + Cetuximab vs. BYL719 + Cetuximab + LGX818	I/II	NCT01719380
Buparlisib (BKM120)	PI3K	Wild-type RAS advanced or metastatic CRC	Panitumumab + BKM120	I/II	NCT01591421
Gedatolisib (PF05212384)	PI3K/mTOR	mCRC	Gedatolisib + FOLFIRI vs. FOLFIRI + Bevacizumab	I/II	NCT01937715
Nab-rapamycin (ABI-009)	mTOR	1st line advanced or metastatic CRC	ABI-009 + FOLFOX + bevacizumab	I/II	NCT03439462
Everolimus (RAD001)	mTOR	2nd line mCRC	Irinotecan + Cetuximab ± Everolimus	I/II	NCT00522665
ONC201	AKT/ERK	MSS mCRC	ONC201 + Nivolumab	I/II	NCT03791398
MK2206	AKT	WT k-RAS/mutated PIK3CA mCRC	MK2206	II	NCT01186705
TTI-101	STAT3	Advanced CRC	TTI-101	I	NCT03195699
Ruxolitinib	JAK/STAT3	RAS mutant advanced CRC and pancreatic cancer	Ruxolitinib + trametinib	I	NCT04303403

mCRC: metastatic colorectal cancer; FOLFIRI: irinotecan in combination with 5-fluorouracil and folinic acid; MSS: microsatellite stability; FOLFOX: leucovorin calcium, fluorouracil, and oxaliplatin; KRAS: Kirsten rat sarcoma viral oncogene; WT Kras: wild-type Kras; HER: human epidermal growth factor receptor; EGFR: epidermal growth factor receptor; BRAF: v-raf murine sarcoma viral oncogene homolog B1; PIK3: phosphatidylinositol 3-kinase; mTOR: mammalian target of rapamycin. JAK: Janus kinase; STAT3: signal transducer and activator of transcription 3.

## Abbreviations

APC: Adenomatous polyposis coli
AREG: Amphiregulin
BSC: Best supportive care
CapeIRI: Capecitabine and irinotecan
CIMP: CpG Island Methylator Phenotype
CIN: Chromosomal instability
c-MET: Mesenchymal epithelial transition factor
CMS: Consensus molecular subtype
CRC: Colorectal cancer
dMMR: Deficient mismatch repair
EGFR: Epidermal growth factor receptor
EMA: European medicines agency
EMT: Epithelial-to-mesenchymal transition
ERBB: Erythroblastosis oncogene B
EREG: Epiregulin
FAP: Familial adenomatous polyposis
FDA: Food drug administration
FGFR: Fibroblast growth factor receptor
FOLFIRI: Fluorouracil, leucovorin and irinotecan
FOLFOX: Leucovorin calcium, fluorouracil, and oxaliplatin
FU: Fluorouracil
HER: Human epidermal growth factor receptor
HGF: Hepatocyte growth factor
HNPCC: Hereditary nonpolyposis colorectal cancer
HR: Hazard ratio
IDLs: Insertion-deletion loops
IFL: Irinotecan, fluorouracil and leucovorin
IGF-1R: Insulin-like growth factor 1 receptor
IgG: Immunoglobulin G
IHC: Immunohistochemistry
JPS: Juvenile polyposis syndrome
KRAS: Kirsten rat sarcoma viral oncogene
LOH: Loss of heterozygosity
LV: Leucovorin
mAbs: Monoclonal antibodies
MAP: MYH-associated polyposis
MAPK: Mitogen-activated protein kinase
mCRC: Metastatic CRC
MMR: Mismatch repair
MSI: Microsatellite instability
MSI-H: Microsatellite instability high
OR: Odds ratio
ORR: Overall response rate
OS: Overall survival
PDGFR: Platelet-derived growth factor receptor
PFS: Progression-free survival
PGF: Placental growth factor
PJS: Peutz Jeghers
POLE: Polymerase-*ε*
RFS: Relapse-free survival
RR: Response rate
RTKs: Receptor tyrosine kinases
SCNAs: Somatic copy number alterations
TCGA: The cancer genome atlasTGF:
TGF: Transforming growth factor
TTP: Time to progression
VEGF: Vascular endothelial growth factor
VEGFR: Vascular endothelial growth factor receptor
XELOX: Capecitabine (xeloda) and oxaliplatin.
